# The Effectiveness of the Blended Learning in Conservative Dentistry with Endodontics on the Basis of the Survey among 4th-Year Students during the COVID-19 Pandemic

**DOI:** 10.3390/ijerph18094555

**Published:** 2021-04-25

**Authors:** Kacper Nijakowski, Anna Lehmann, Jakub Zdrojewski, Monika Nowak, Anna Surdacka

**Affiliations:** 1 Department of Conservative Dentistry and Endodontics, Poznan University of Medical Sciences, 60-812 Poznan, Poland; annalehmann@ump.edu.pl (A.L.); annasurd@ump.edu.pl (A.S.); 2 Student’s Scientific Group in Department of Conservative Dentistry and Endodontics, Poznan University of Medical Sciences, 60-812 Poznan, Poland; jakub-zdrojewski11@wp.pl (J.Z.); monikaanowak@opoczta.pl (M.N.)

**Keywords:** COVID-19, SARS-CoV-2, pandemic, dental education, e-learning, blended-learning, conservative dentistry, endodontics, dentistry, dental procedures

## Abstract

The COVID-19 pandemic has undoubtedly affected education at all levels, including medical and dental education. Our study aimed to assess the effectiveness of the blended learning in conservative dentistry with endodontics. The students had theoretical classes in a remote form (using the e-learning portal and Teams communicator) and practical classes with the participation of patients in the appropriate sanitary regime. The author’s survey was conducted among fourth-year dental students. The online questionnaire consisted of 5 parts: self-evaluation, evaluation of theoretical e-learning classes, evaluation of practical clinical classes, evaluation of safety, and evaluation of performed blended learning. The majority of respondents declared that their learning effectiveness increased during the pandemic. Most surveyed students preferred remote learning in asynchronous form (e-learning portals) to synchronous form (virtual meetings in real-time). All respondents described the provided personal protective equipment as sufficient or even as excessive. Our students were very satisfied with the proposed blended-learning model and would like to continue it even after the pandemic has ended. Among the advantages, they particularly mentioned the increase in efficiency and the individualised pace of learning, while the disadvantage was the limitation of social contacts. The appropriate use of modern technology can effectively revolutionise dental education.

## 1. Introduction

SARS-CoV-2 is a virus detected in Wuhan, Hubei Province in China at the end of 2019 [1]. Since the day of the first report of infection, every day, new cases have been emerging and the new pathogen has spread all over the world. On 11th March 2020, the World Health Organization declared that the coronavirus causing COVID-19 is referred to as the pandemic [2]. The infection manifests in high fever, dry cough, difficulty breathing, tiredness, and muscle pain as well as loss of taste and smell. The most dangerous consequence of SARS-CoV-2 infection is it may lead to acute respiratory failure, and the treatment is based on alleviating the symptoms [3].

The lack of knowledge of the virus transmission and how to prevent the infection was an additional danger. The only way of reducing transmission was to keep social distance [4]. Therefore, because of the rapid increase in infection cases, most countries decided to introduce lockdown, which was supposed to restrict social activities only to meet basic life needs. On 24th February in Italy and on 11th March in Poland, the national governments implemented several restrictions, such as the closure of schools and universities. Every class that had taken place at the University, such as seminars, workshops, laboratories, and classes with patients, were cancelled [5,6].

The epidemiological situation in the world and Poland has posed a great challenge for dental education teachers as well as students. At the beginning of the COVID-19 pandemic, the New York Times published an article describing dentists as the most exposed group to the SARS-CoV-2 [7]. While operating basic tools such as high-speed turbine, spray handpiece, and ultrasonic scaler, the generated aerosol significantly increases the risk of infection [8]. It is extremely difficult to balance safety with development and obtaining practical skills, which are highly required in the dental profession [9,10]. In the case of doctors and students who are conducting aerosol-generating procedures, their personal protective equipment (PPE) should be face masks, disposable gloves, a face shield, goggles, and a protective suit [11,12]. Special face masks with N95 filter (with 95% filtration efficiency) are recommended by the US National Institute for Occupational Safety (NIOSH); according to European standards, face masks FFP2 have comparable effectiveness to N95 face masks. Eventually, double surgical masks were also used to protect air-passages against aerosol [13,14].

The only sensible solution was distance teaching and implementing e-learning platforms. E-learning is based on using online tools such as academic e-learning platforms for playing recorded lectures and seminars, doing self-tests which can monitor progress in studies, and MS Teams programs enabling holding real-time, online meetings instead of face-to-face ones [15]. The communication between academic tutors and students was limited to conversations on internet communicators, e-mails, and educational platforms. The new reality was a great challenge to IT specialists, teachers, and students. At the Poznan University of Medical Sciences (PUMS), some academic coordinators completed their training in order to improve their IT skills and prepare e-learning materials to effectively educate students of medical sciences and be able to use them in the next academic year [16]. 

Conservative dentistry with endodontics is one of the main fields of dentistry. At the PUMS, for fourth-year students, it is a leading compulsory subject of 210 teaching hours (converted into 10 ECTS [European Credit Transfer System] points). Conservative procedures are essential skills that students should acquire in their undergraduate education. Unfortunately, the pandemic caused changes in the spectrum of provided dental services. As an example of our University Centre of Dentistry and Specialised Medicine in Poznan, during the pandemic period, especially the first wave, the performance of conservative procedures decreased and the performance of surgical procedures increased significantly [17]. As in the case of dental care, the question of how to treat should be asked; in the case of dental education, the question of how to teach effectively should be answered.

After several months of dealing with the pandemic, most of the universities in the world were challenged to organize the 2020/2021 academic year. At the beginning of this academic year, Poland was facing the second wave of the COVID-19 pandemic. On 7th November 2020, there was reported a record number of coronavirus cases, which was 27,875 [data from official Polish government site]. Moreover, on 27th December 2020, Poland started a National Vaccination Programme whereby medical practitioners, including medical and dental students, were vaccinated in the first phase, so-called Stage 0. This programme would increase the safety of medical practitioners and patients and give hope of a slow return to normal life.

The COVID-19 pandemic changed the stomatology and the way of teaching it that might be continued in the post-pandemic time. Blended learning has been introduced and has become the optimal solution in future doctors’ education. It combines e-learning activities allowing the gain of essential theoretical knowledge and clinical workshops in traditional form (while keeping a full sanitary regime) enabling the gaining of all practical skills. Moreover, blended learning also allows increasing communication with module tutors through instant messengers in order to obtain answers to issues discussed during the online classes. In this teaching model, exams and tests can be performed by sOLAT (academic e-learning portal at PUMS), MS Teams, and Socrative platforms. The theoretical examinations are constructed in the form of multiple/single-choice questions, true-or-false questions, and/or matching description with the pictures or the clinical cases [16]. During the examination session, the students should be in their camera range for the whole time [18]. In turn, clinical workshops have been reorganized in order to meet the new sanitary guidelines for dental treatment. This was to keep doctors as well as their patients safe [19,20].

Our study aimed to assess the effectiveness of blended learning in conservative dentistry with endodontics, especially on the basis of the survey among fourth-year students during the COVID-19 pandemic.

## 2. Materials and Methods

The Poznan University of Medical Sciences is the leading academic centre in Poland which allowed students to continue dental clinicals during the second wave of the COVID-19 pandemic. Safe conduct of classes was possible thanks to the observance of the sanitary-epidemiological regime, provision of advanced personal protective equipment, and development of efficient preventive procedures in case of infection among students or academic teachers. It should be emphasised that 80% of the academic hours in the subject curriculum are dedicated to practical classes. In turn, thanks to the virtual experience gained in the first wave of the pandemic, it was possible to organise an e-learning model during the second wave. The seminars were made available on the university’s portal in the form of recorded presentations and self-tests of the covered material, and each thematic block ended with a discussion meeting using Teams messenger. Over 60% of theoretical classes were performed in asynchronous form.

The study design was defined as educational action research. Our survey was conducted among fourth-year dental students who all participated in the blended-learning course. Students were given the link to a Google form (composed of closed and open questions) to fill out. The 39-item author’s questionnaire consisted of 5 parts: self-evaluation, evaluation of theoretical e-learning classes, evaluation of practical clinical classes, evaluation of safety during COVID-19 pandemic, and evaluation of performed blended learning. Most of the questions were based on a 5-point Likert scale or a dichotomous scale. The survey form was pre-validated on a group of 5 volunteers. The evaluation questionnaire (translated into English) is provided as Supplementary Material.

The survey results were presented in tables and graphs. For analysing part of the questions, the respondents were divided into two groups—students who had the clinical exercises in the third year and students who took the classes online only. The answers were compared using the Mann–Whitney’s test or the Wilcoxon’s test. Multidimensional correspondence analysis was used to assess the relationship between potential advantages/disadvantages of blended learning and its evaluation. 

Moreover, the base of patients participating in fourth-year classes in the Department of Conservative Dentistry and Endodontics from two winter semesters—pre-pandemic (October 2019–January 2020) and pandemic (October 2020–January 2021)—was evaluated. Selected procedures in conservative dentistry with endodontics were analysed in detail. The number of these individual services was standardised against the number of practising student pairs (per day) in a given block of classes. Each block had 4-h clinical classes every day for 4 weeks with two student groups (each dental year has six student groups in total). Comparisons were made between the performed procedures in the pre-pandemic and the pandemic periods using the Mann–Whitney test.

All statistical analyses were performed using the Statistica 13.3 (StatSoft, Cracow, Poland). The significance level was estimated at α = 0.05.

## 3. Results

**Figure 1:**
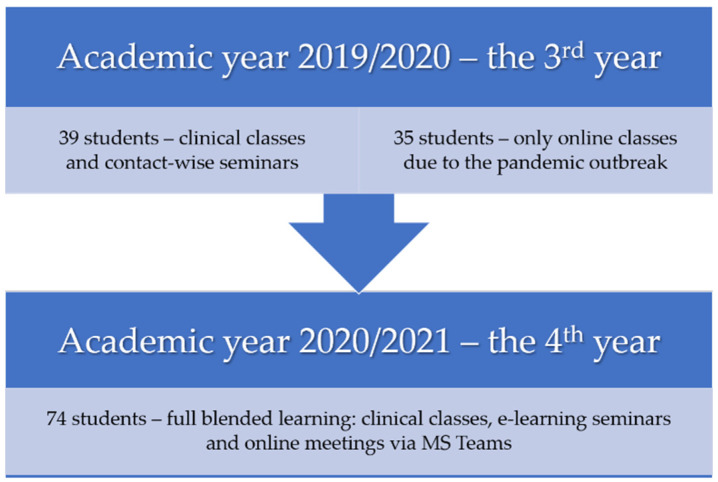
Graph presenting the structure of the study groups.

The final return rate of the questionnaires was 90.2% (74/82). Among the respondents, there were 57 women (77.0%) and 17 men (23.0%). The median age of the study group was 23 years. Thirty-nine surveyed students had clinicals and contact-wise seminars before the pandemic and thirty-five of them had only online classes last academic year (Figure 1).

### 3.1. Self-Evaluation

Table 1 presents the results of the answers concerning the self-assessment of theoretical knowledge, practical skills, and interpersonal skills acquired during the third (retrospectively) and fourth-year courses in conservative dentistry with endodontics. There is a significant increase in scores in all aspects between the third and fourth year regardless of the classes taken before the pandemic. 

Lower self-assessment of practical and interpersonal skills was declared after the third year by students who did not have clinical classes. In turn, after the winter semester of the fourth year, this group rated themselves much higher, especially in the aspect of communication with patients and performing dental procedures.

**Figure 2:**
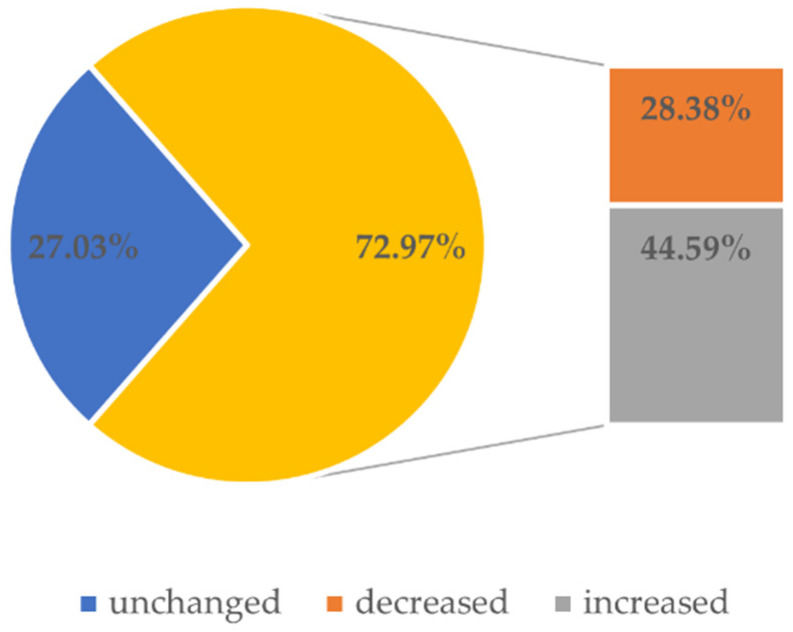
Graph presenting the impact of the COVID-19 pandemic on learning effectiveness.

Figure 2 shows how the surveyed students assessed the change in their academic performance under the impact of the COVID-19 pandemic. Surprisingly, a large proportion of students indicated that their overall learning effectiveness increased during the pandemic. Moreover, one-third of respondents reported sharing learning with friends before the pandemic, with only one quarter continuing to do so in the pandemic period. It was stated that the closure of the academic library building was the main reason for this restriction.

### 3.2. Evaluation of Theoretical e-Learning Classes

Evaluation of particular aspects of theoretical teaching in the form of e-learning is presented in Table 2. All of the components were rated very highly, both by groups who took the seminars contact-wise and by groups who took these classes online last academic year. 

However, compared to the third year, the surveyed students rated better, especially the following criteria: quality of presentations (81.1%) and commitment of the teachers (73.0%). For the remaining issues, the responses were spread more or less equally between better and no change. 

**Figure 3:**
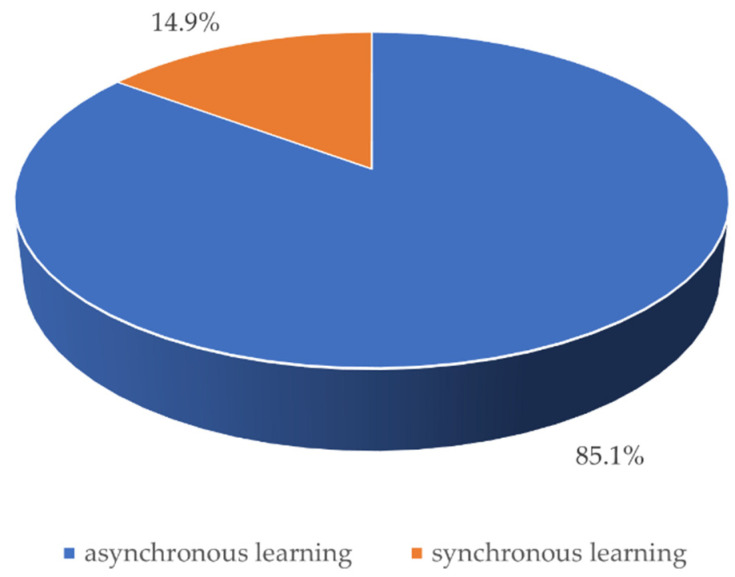
Graph presenting the forms of remote learning preferred by respondents.

As far as the choice between the forms of remote learning is concerned, as many as 85.1% of respondents opted for asynchronous learning (on the e-learning platform) and only 14.9% for synchronous learning (online meetings via MS Teams)—Figure 3. Among the advantages of asynchronous learning, students mentioned the possibility of multiple playbacks of presentations, the ability to listen to materials at any time, as well as the ability to work at their own pace. On the other hand, in synchronous learning, the most common advantage was the opportunity to engage in face-to-face discussion in a real meeting time.

### 3.3. Evaluation of Practical Clinical Classes

Table 3 shows the results of the evaluation of analogous aspects concerning clinical classes. Clinicals were rated significantly higher by groups that already had classes before the pandemic. They particularly valued their own involvement in the classes (which correlated with the rating of the involvement of their tutors, higher than in the previous year), as well as the spectrum of performed procedures. However, it should be explained that in contrast to theoretical classes conducted by one team, clinical classes in particular blocks were conducted by different dental teachers preferring various types of procedures, which undoubtedly could have affected the assessment of the quality of classes. Section 3.6 compares the spectra of performed procedures before and during the COVID-19 pandemic in each block of the winter semester.

### 3.4. Evaluation of Safety during COVID-19 Pandemic

Among 4th-year students, there were only 12 cases of referral to quarantine or isolation (16.2% of respondents), particularly in October 2020. Only three women did not initially declare their willingness to be vaccinated (4.1% of surveyed students).

**Figure 4:**
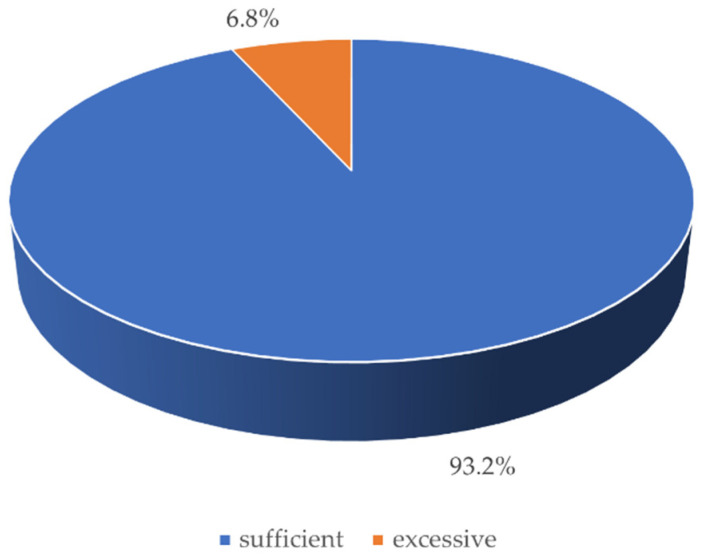
Graph presenting the evaluation of the provided personal protective equipment.

Before the start of clinical activities, concerns about SARS-CoV-2 infection were shown by 13 students (17. 6%). However, during clinicals, the safety of students was rated highly (M [Q_1_, Q_3_] was 5.0 [4.0, 5.0] on a 5-point scale). Patient safety during teaching procedures was similarly assessed (M [Q_1_, Q_3_] = 5.0 [4.0, 5.0]). Moreover, 93.2% of the respondents described the provided personal protective equipment as sufficient and the remainder even as excessive (Figure 4).

For screening prior to attendance, rapid antigen tests would be recommended for students and assistants as well as for patients by 43.2% and 40.5% of respondents, respectively. The majority of students indicated that teachers and students themselves should be tested a minimum of once a week or as soon as symptoms occur. In turn, as to the introduction of compulsory vaccinations, the surveyed students were divided roughly 50-50. Compulsory vaccination would increase the safety of patients, doctors, and other employees of Centre, as well as limit the transmission of the virus, enabling the return to normality. However, another part of the respondents thought that vaccination should be voluntary, but as future doctors, they should promote proper patterns among society.

### 3.5. Evaluation of Performed Blended Learning

**Figure 5:**
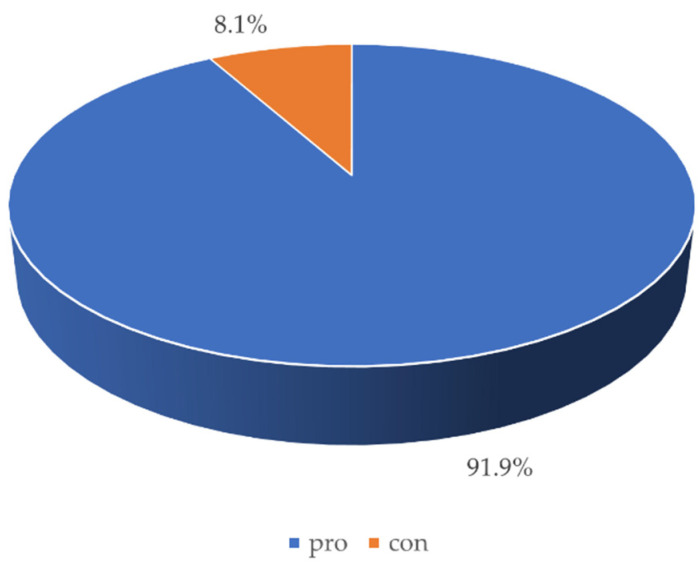
Graph presenting the student interest in continuing blended learning after the COVID-19 pandemic.

The blended-learning model proposed in our department during the fourth year was evaluated very highly (M [Q_1_, Q_3_] was 5.0 [4.0, 5.0] on a 5-point scale). Nearly 90% of the surveyed students stated that the performed form of teaching in conservative dentistry with endodontics was better than in other dental academic departments. The majority of the respondents emphasised, in particular, the thorough preparation of the seminars (in the form of recorded presentations and self-tests with the possibility to return to the discussed contents), the extraordinary commitment of the lecturers, the detailed and precise teaching plan, as well as the generally perfect organisation. Moreover, more than 90% of respondents declared that they would like to continue the blended learning even after the COVID-19 pandemic ends (Figure 5).

**Figure 6:**
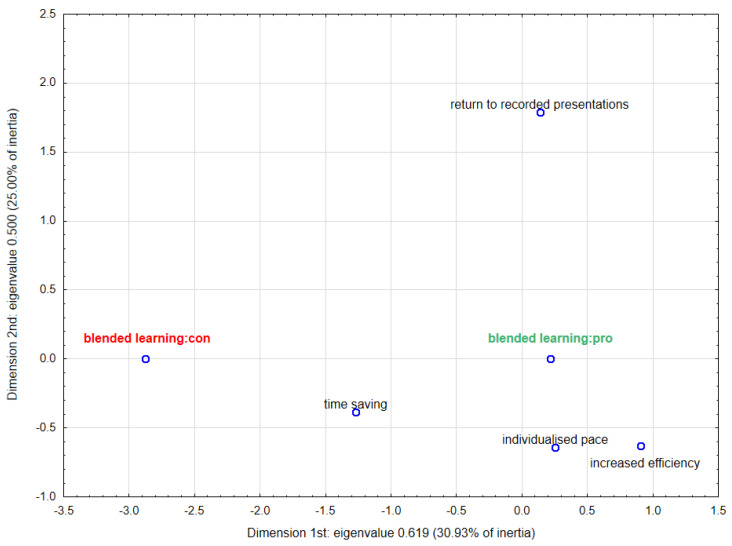
Two-dimensional plot for multidimensional correspondence analysis—advantages of blended learning.

**Figure 7:**
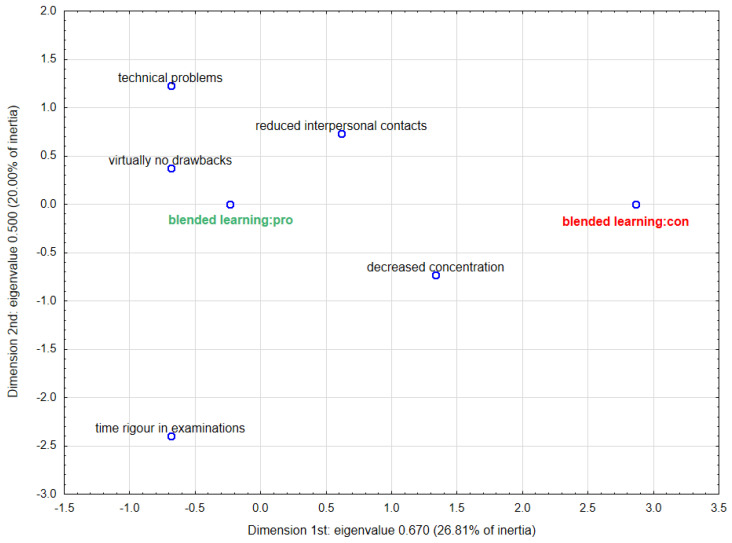
Two-dimensional plot for multidimensional correspondence analysis—disadvantages of blended learning.

In order to graphically illustrate the relationship between the advantages or disadvantages of blended learning and the desire to continue it after the COVID-19 pandemic, a multidimensional correspondence analysis (MCA) was conducted. Based on the scree plots (Figures S1 and S2), it was decided to choose two-dimensional analysis as the best description of the examined phenomenon. Figure 6 and Figure 7 show the results of MCA in two dimensions. Tables S1 and S2 contain parameters characterising the determined points. In the case of the first plot showing the advantages (Figure 6), the points representing increased efficiency and individualised learning pace are concentrated closest to approval for blended learning, which indicates a stronger link for these pros. The second plot presenting the disadvantages (Figure 7) confirms the relationship between aversion to blended learning and reduced interpersonal contacts as well as decreased concentration (the smaller angles with vertex at the beginning of the coordinate system). On the same basis, it can be concluded that the majority of those in favour of continuing this form of education perceived virtually no drawbacks.

### 3.6. Comparison of the Spectrum of Selected Procedures

Table 4 presents the results of comparing the spectrum of selected procedures performed before and during the pandemic based on standardised values against the number of practising student pairs in a given block of classes. During the COVID-19 pandemic, the number of root canal fillings decreased, while the number of tooth filling increased. However, it should be emphasised that no statistically significant differences were found. Importantly, in individual months of both winter semesters, clinical classes were conducted by the same teams of dental teachers.

## 4. Discussion

The Association of Dental Education in Europe carried out the survey to determine the initial response of European dental schools to the COVID-19 crisis [21]. The questionnaire reported five areas: clinical activities, non-clinical activities, assessment forms, pastoral support, and future implications. The clinical work was mainly performed by the academic staff with the assistance of postgraduate students. In turn, undergraduate students as volunteers were asked to help during non-clinical activities. All schools reported limited access to academic buildings and providing distance learning. The most common forms of online learning were live or streamed videos, links to other electronic materials, and virtual meetings. The lockdown also resulted in the deferral of formative and summative assessments. The majority of examinations were held online. Most schools postponed the assessment of practical skills. During this challenging time of isolation, psychological help was offered to students through virtual messengers. It should be emphasised that dental education differs significantly from medical education due to the greater emphasis on manual skills training. Therefore, the pandemic has had a very strong impact on the instruction of dental students, and it is necessary to implement effective educational alternatives before the return to the new normality.

During the first wave of the pandemic in March 2020, our department was the only dental one that immediately switched to e-learning based on clinical cases for the fourth-year students. The following virtual solutions were used: sOLAT (as the university’s e-learning portal), MS Teams (for synchronous/real-time communication, mainly summing up a completed online study session and discussion of all the preceding tasks), and Web 2.0 tools (such as Socrative or Kahoot for diagnostic quizzes based on intraoral and radiological images). Our e-learning course consisted of the following components: forum and notifications for asynchronous communication (organisational messages, daily work schedules, pass requirements, etc.), educational materials (seminar presentations, demonstration videos, and guidelines), project task (open-ended questions related to clinical cases in endodontics, evaluated with individual feedback), practice test for a self-check-in diagnostics/therapy (with true/false questions), and interface ask a teacher (for quick individual student-teacher communication). Dentists as academic teachers successfully met the new challenges, which was confirmed by the feedback of students whose clinical classes had been suddenly interrupted overnight by the pandemic [16].

In our study, for theoretical content, most respondents preferred remote learning in the asynchronous form (i.e., e-learning portals) to synchronous form (i.e., virtual meetings in real-time). They considered the possibility of individualisation of the time and pace of learning as the main advantage.

In contrast, the survey conducted by Amir et al. [22] demonstrated that only nearly half of the students advocated distance learning over classroom learning. This finding was mainly due to technical problems and lack of permanent access to the Internet among Indonesian students. Nevertheless, they were not against the gradual introduction of technological novelties into dental education. Most respondents (87.4%) preferred synchronized learning sessions for group discussions.

Moreover, Chang et al. [18] tried to exchange the experience of dental educators from different countries during the COVID-19 pandemic. The results showed that almost all lectures were held online. Virtual meetings were proceeded using various applications such as Zoom, Google Meet, Skype, and Microsoft Teams. Some dental schools in the USA used special browsers during exams to prevent students from cheating by searching for answers on the Internet. In most countries, both pre-clinical and clinical activities were suspended during the first wave of the pandemic. The authors believe that education needs to be made more attractive through the introduction of innovative solutions, the potential of which has been demonstrated by the pandemic. Academic teachers should not be afraid to move away from traditional teaching methods but should show more flexibility towards current paths.

In turn, Van Doren et al. [23] evaluated how the pandemic had affected dental education from the students’ point of view. Most students felt that the teaching of practical skills (both pre-clinical and clinical) had deteriorated. The majority of respondents stressed the lack of opportunities to develop manual work experience. They appreciated the possibility of virtual discussions on clinical cases, as it taught critical thinking, although they believed that it could not replace direct contact with patients. The presentation of instructional videos demonstrating specific treatments was considered a good solution, even after the pandemic has ended.

The majority of our surveyed students indicated that their learning efficiency increased during the pandemic. Additionally, they declared that the quality of teaching this subject increased in comparison to the pre-pandemic period. In the innovative blended-learning model proposed by us, the questionnaire respondents appreciated, above all, very high involvement and excellent organisation.

Similarly, in the study of Turkyilmaz et al. [24], the factors such as organization and logic of content were the most important advantages of e-learning. The surveyed students declared that such a form of teaching definitely increased the understanding of the presented clinical content. Moreover, they observed that younger teachers are more inclined to enrich their courses with e-learning elements and conduct efficient virtual communication.

Liu et al. [25] compared the forms of dental education during two periods: the pre-COVID-19 pandemic (ten weeks from November 2019 to January 2020) and the COVID-19 pandemic (two weeks in February 2020). Among the 21 evaluated academic institutions, only 6 had online classes prior to the pandemic outbreak. Surprisingly, 33 online courses were conducted during the entire control period and 119 during the initial two weeks of the pandemic. According to teaching live online classes, the pandemic has significantly increased the proportion in provided working time from 6.1% to 46.2%. The authors noted that distance learning is a challenging process, requiring teachers to pay more attention to the needs and engagement of students, with whom motivating interaction is key to success.

In our survey, the blended learning was rated very highly. Most students would like to continue this form of education even after the COVID-19 pandemic has ended. Among the advantages, they emphasised the increase in learning efficiency and individual pace of work with the provided materials. Limited interpersonal contacts were stated as one of the disadvantages of distance learning. In the other study [26], dental students also positively evaluated the used blended-learning model, particularly emphasizing the advantage of self-regulated learning.

Al-Azzam et al. [27] tried to determine the factors which significantly influence the preference for virtual learning among the students of dental and medical college (especially first-year ones). Based on the survey responses, they constructed the logistic regression model with high prediction quality. Among the relevant factors, the authors found accessibility of online tools, class engagement, class attendance, time-saving, as well as GPA (Grade Point Average) change and anxiety level during the COVID-19 outbreak. For example, the calculated odds that the student prefers virtual courses are above four times higher for the student who feels more engaged in this kind of class. However, only 32% of respondents preferred virtual learning compared to on campus.

The study of Alsoufi et al. [28] aimed to determine the attitude of medical students towards remote education. More than half of the respondents thought that recorded lectures were better and could replace traditional lectures. A similar proportion said that it was possible to have effective discussions via instant messaging. However, a large part of students disagreed with the statement that e-learning can be used to teach clinical aspects. Most students used the Internet for discussions, but the minority used it additionally for e-learning courses during the COVID-19 pandemic.

Similarly, Schlenz et al. [29] assessed the students’ and lecturers’ perspectives on the implementation of online learning during the COVID-19 pandemic. The overall assessment of this learning form by both study groups was positive, and they wanted to continue it partly in the future. On the question of the optimal amount of classes conducted remotely, students differed significantly from teachers, suggesting as many as over half of the classes compared to over one-third. Most students learned at home using a laptop. In contrast, academic teachers most often conducted their classes from the office. In online learning, students did not feel disappointed and appreciated modernity while having some fun and saving time. For teachers, the most important aspects were the ease of participating and time effort.

Regarding safety in clinical classes, most of our students did not feel apprehensive about participating. They rated the proposed personal protective equipment as sufficient or excessive. There was no consensus among respondents on screening rapid antigen tests and mandatory vaccination.

Loch et al. [30] investigated final-year dental students and clinical staff perceptions of health risks and impacts on clinical competence in mid-March 2020 when clinical activities were still operational at the University of Otago. The majority of respondents felt that they risked their health with the clinical work during the COVID-19 outbreak, which was associated with a significant increase in stress levels. The additional concern for the high percentage of both students and clinical teachers was the potential risk of SARS-CoV-2 transmission from patients in classes to their family members or roommates. A significant proportion of respondents considered that personal protective equipment to date was insufficient and stressed the need to triage patients, postpone visits for symptoms of respiratory infections, and emphasize return from countries with high coronavirus incidence. They also suggested implementing preventive procedures such as introducing N95 masks, improving the ventilation of clinical rooms, rinsing the oral cavity with antimicrobial liquids before treatment, and using rubber dam isolation and a high-volume suction system. Procedures generating aerosols have been clearly identified as a major contributing factor in cross-infection. Both students and clinical staff indicated online discussion based on clinical cases as the preferred alternative dental education method during the suspension of clinical classes due to the COVID-19 pandemic. The surveyed students proposed to organise additional practical classes after the pandemic in order to make up for the time in clinical work.

The study of Tuells et al. [31] aimed to assess the prevalence of immunity against SARS-CoV-2 among the students and the academic staff using a rapid immunoassay test in July 2020. The determined seroprevalence was 2.64% (39/1479). In relation to SARS-CoV-2 cases, the following percentages were found: 17.7% paucisymptomatic, 1.3% symptomatic, 5.5% contact with cases, 4.9% confined, and 0.3% PCR positive. As many as 91% of the respondents were interested in being vaccinated against COVID-19.

In addition, Hung et al. [32] evaluated the impact of the COVID-19 pandemic on dental education, especially on dental students’ experiences. Most respondents believed that, above all, adequate social distance could prevent the spread of the coronavirus. Additionally, more than one-third of the surveyed students indicated that testing for coronavirus should be performed. Younger students showed more concern about their mental health due to increased stress. More than two-thirds of respondents felt concerned about the possibility of infection from patients admitted in clinical classes. Nearly half the students reported problems with finding the motivation to study. In turn, virtually all declared a comfortable attitude towards modern technologies and their use during learning. The majority of respondents evaluated the online classes positively; however, only a minority were satisfied with the form of clinical experience providing. The authors suggested that the pandemic crisis will allow enriching dental education with new methods based on modern technologies.

Furthermore, Zis et al. [33] investigated the impact of digital learning on the burnout and overall mental health of medical students during the COVID-19 pandemic. The burnout prevalence did not differ significantly between the pre-COVID and COVID periods. However, the burnout decreased significantly in fourth-year students and increased in sixth-year students. These findings were explained by the authors that the final-year students were concerned about the lack of scheduled practicals just before graduation. On the other hand, the surprising improvement in younger students may have been related to less mental stress due to the lack of first clinical classes scheduled intensively during this period of medical studies.

In the case of practical vocational subjects, the essential issue is the teaching of manual skills, which cannot be replaced by the remote learning form. As a result of the well-implemented blended-learning system and the experience gained during the first wave of the pandemic, the number of clinical procedures performed by our students has not decreased, which is suitable for their quality of education. A maintained balance between theoretical classes in the form of e-learning and clinical activities in the sanitary regime allowed an appreciation of involvement by students.

Qutieshat et al. [34] compared the effectiveness of blended learning and traditional methods for fourth-year dental students in two consecutive cohorts in a conservative dentistry course. Blended-learning students achieved significantly higher grades than traditionally taught students. Moreover, most respondents were satisfied or very satisfied with the blended-learning model. Surprisingly, the study of Maresca et al. [35] showed that in preclinical endodontic education, students could also acquire practical skills even better than those experiencing traditional education. The achieved results correlated with the positive opinions of students about the innovative form of teaching.

Our study was limited to one academic centre and one year where we introduced the complete model of blended learning. Thanks to the experience gained during the first wave of the COVID-19 pandemic, it was possible to prepare it in a way suitable for students. Nevertheless, it seems that the obtained results may reflect the need to change the education manner for young students accustomed to modern technologies. If teaching in this format becomes standard at other universities, regardless of the pandemic conditions, further and broader research would be desirable.

## 5. Conclusions

The COVID-19 pandemic has undoubtedly affected education at all levels, including medical and dental education. Thanks to the possibility of remote learning, theoretical teaching has been carried out continuously using e-learning platforms and instant messengers. In turn, clinical classes conducted in small groups using appropriate personal protective equipment allowed for the safe performance of a wide range of dental procedures on patients. Our students are very positive about the blended-learning model and would like to continue it even after the pandemic. Fortunately, with the development of proper safety rules and the introduction of vaccination, a slow return to the effective training of future doctors and dentists is becoming possible.

## Figures and Tables

**Table 1:** Answers concerning the self-assessment of theoretical knowledge, practical skills, and interpersonal skills acquired during the third and fourth-year courses in conservative dentistry with endodontics (based on the 5-point scale).

		Clinicals before the Pandemic N = 39	No Clinicals before the Pandemic N = 35	*p*-Value
		M (Q_1_–Q_3_)	M (Q_1_–Q_3_)	
Theoretical knowledge	3rd year	3.0 (3.0–4.0)	3.0 (3.0–4.0)	0.702
	4th year	4.0 (4.0–4.0)	4.0 (4.0–4.0)	0.879
	*p*-value	0.001 ^	0.001 ^	
Practical skills	3rd year	3.0 (2.0–4.0)	2.0 (1.0–2.0)	<0.001 *
	4th year	4.0 (3.0–4.0)	3.0 (3.0–4.0)	0.083
	*p*-value	<0.001 ^	<0.001 ^	
Interpersonal skills	3rd year	4.0 (3.0–5.0)	3.0 (2.0–4.0)	0.008 *
	4th year	4.0 (4.0–5.0)	4.0 (4.0–5.0)	0.952
	*p*-value	0.048 ^	<0.001 ^	

* significant difference for *p*-value < 0.05 according to the Mann–Whitney test; ^ significant difference for *p*-value < 0.05 according to the Wilcoxon test.

**Table 2:** Evaluation results of selected e-learning issues (based on the 5-point scale).

	Clinicals before the Pandemic N = 39	No Clinicals before the Pandemic N = 35	*p*-Value
	M (Q_1_–Q_3_)	M (Q_1_–Q_3_)	
Quality of recorded presentations	5.0 (5.0–5.0)	5.0 (5.0–5.0)	0.378
Preparation for self-tests	4.0 (4.0–4.0)	4.0 (4.0–5.0)	0.561
Commitment to learning	4.0 (4.0–5.0)	4.0 (4.0–5.0)	0.763
Time spent learning	4.0 (3.0–4.0)	4.0 (4.0–4.0)	0.751
Commitment of the teachers	5.0 (5.0–5.0)	5.0 (5.0–5.0)	0.787
Spectrum of discussed issues	5.0 (4.0–5.0)	5.0 (4.0–5.0)	0.469

* significant difference for *p*-value < 0.05 according to the Mann–Whitney test.

**Table 3:** Evaluation results of selected clinical issues (based on the 5-point scale).

	Clinicals before the Pandemic N = 39	No Clinicals before the Pandemic N = 35	*p*-Value
	M (Q_1_–Q_3_)	M (Q_1_–Q_3_)	
Quality of conducted clinicals	5.0 (4.0–5.0)	4.0 (4.0–5.0)	0.051
Preparation for clinicals	4.0 (4.0–5.0)	4.0 (4.0–4.0)	0.354
Commitment to clinical work	5.0 (4.0–5.0)	4.0 (4.0–5.0)	0.040 *
Time spent preparing for clinicals	4.0 (4.0–5.0)	4.0 (3.0–4.0)	0.264
Commitment of the teachers	5.0 (4.0–5.0)	5.0 (4.0–5.0)	0.073
Spectrum of performed procedures	4.0 (3.0–5.0)	3.0 (3.0–4.0)	0.034 *

* significant difference for *p*-value < 0.05 according to the Mann–Whitney test.

**Table 4:** Standardised numbers of selected procedures performed before and during the COVID-19 pandemic in winter semesters by successive blocks of fourth-year dental students in the Department of Conservative Dentistry and Endodontics at PUMS (Poland).

	Before Pandemic			During Pandemic			
Procedure	Oct-19	Nov-19	Dec-19/Jan-20	Oct-20	Nov-20	Dec-20/Jan-21	*p*-Value
Dental examination	10.33	10.25	8.47	12.75	9.88	11.25	0.383
Periapical X-ray	3.73	5.50	4.40	3.50	6.25	2.75	0.663
**Conservative dentistry**							
Temporary filling	2.67	4.25	4.07	1.63	1.38	1.75	0.081
Single-surface filling	12.53	11.08	9.67	11.50	14.00	9.88	0.663
Two-surface filling	7.33	6.83	8.87	8.25	9.38	8.50	0.383
Multi-surface filling	0.73	0.75	1.07	1.50	2.38	1.38	0.081
Total filled teeth	20.60	18.67	19.60	21.25	25.75	19.75	0.190
**Endodontics**							
Intervention procedure	0.47	0.58	0.80	0.38	0.38	0.50	0.184
Intracanal dressing	0.27	0.92	0.27	0.00	0.38	0.00	0.369
Single-rooted filling	0.27	2.17	0.73	0.25	1.38	0.38	0.663
Two-rooted filling	0.13	0.25	0.47	0.00	0.38	0.00	0.376
Multi-rooted filling	0.33	0.58	0.60	0.00	0.38	0.13	0.190
Total root canal filling	1.53	4.41	3.47	0.25	3.25	0.75	0.190
Scaling (quadrant)	24.40	12.50	12.33	21.25	14.13	20.00	0.663
Gingival pocket rinsing	0.60	0.67	0.27	0.00	0.63	0.25	0.383
Tooth extraction	0.00	0.33	0.13	0.25	0.88	0.25	0.376

* significant difference for *p*-value < 0.05 according to the Mann–Whitney test.

## Supplementary Materials

The following are available online at https://www.mdpi.com/article/10.3390/ijerph18094555/s1, Translated version of the questionnaire, Figure S1: Scree plot for multidimensional correspondence analysis—advantages of blended learning, Figure S2: Scree plot for multidimensional correspondence analysis—disadvantages of blended learning, Table S1: Parameters of determined points in multidimensional correspondence analysis—advantages of blended learning, Table S2: Parameters of determined points in multidimensional correspondence analysis—disadvantages of blended learning.
