# Supplementary material for: The Effectiveness of the Blended Learning in Conservative Dentistry with Endodontics on the Basis of the Survey among 4th-Year Students during the COVID-19 Pandemic
Source: Int J Environ Res Public Health. 2021 Apr 25;18(9):4555. doi: 10.3390/ijerph18094555 (PMC8123304; doi:10.3390/ijerph18094555)
Supplement: Supplementary file 1 [file ijerph-18-04555-s001.zip › ijerph-1178410-supplementary.pdf]

The effectiveness of the blended learning in conservative dentistry with endodontics on the basis of the survey among 4th-year students during the COVID-19 pandemic *(translated version of the questionnaire)*

**Required*

1. Gender *
Choose only one answer
Female/Male

2. Age

3. Did you participate in the contact-wise clinical classes in conservative dentistry with endodontics in the third year? *
Choose only one answer
Yes/No

4. Did you have to quarantine or isolate yourself during the pandemic due to coronavirus infection (your own or someone close to you)? *
Choose only one answer
Yes/No

5. Do you plan to take the suggested vaccinations? *
Choose only one answer
Yes/No

Self-evaluation

6. On a scale from 1 to 5 (1 the lowest, 5 the highest), how would you rate: *
Select only one answer in a row

	1	2	3	4	5
Your theoretical knowledge after the 3rd year?					
Your practical skills after the 3rd year?					
Your interpersonal skills after the 3rd year?					
Your theoretical knowledge after the first semester of the 4th year?					
Your practical skills after the first semester of the 4th year?					
Your interpersonal skills after the first semester of the 4th year?					

7. Has the effectiveness of your learning changed during the pandemic? *
Choose only one answer
Yes *Go to the 8th question*
No *Go to the 10th question*

Self-evaluation (continued)

8. How has your learning effectiveness changed during the pandemic? *
Choose only one answer
Increased/decreased

9. Why? *

Self-evaluation (continued)

10. Did you learn with friends during the pre-pandemic period? *

Choose only one answer

Yes *Go to the 11th question*

No *Go to the 13th question*

11. Do you still learn with friends? *

Choose only one answer

Yes/No

12. What has changed in this regard?

Evaluation of theoretical e-learning classes in the 4th year in conservative dentistry with endodontics

13. On a scale from 1 to 5 (1 the lowest, 5 the highest), how would you rate: *

Select only one answer in a row

	1	2	3	4	5
quality of recorded presentations?					
preparation for self-tests?					
commitment to learning?					
time spent on learning?					
commitment of the teachers?					
spectrum of discussed issues?					

14. How do you rate compared to traditional classroom-based seminars: *

Select only one answer in a row

	Better	Comparatively	Worse
quality of recorded presentations?			
preparation for self-tests?			
commitment to learning?			
time spent on learning?			
commitment of the teachers?			
spectrum of discussed issues?			

15. Which of the remote classes do you find more beneficial? *

Choose only one answer

the e-learning platform sOLAT/online meetings via MS Teams

16. Why? *

17. Did you feel any deficiencies in the content of the seminars? *

Choose only one answer

Yes/No

18. If so, what kind?

Evaluation of practical clinical classes in the 4th year in conservative dentistry with endodontics

19. On a scale from 1 to 5 (1 the lowest, 5 the highest), how would you rate: *
Select only one answer in a row

	1	2	3	4	5
quality of conducted clinicals?					
preparation for clinicals?					
commitment to clinical work?					
time spent on preparing for clinicals?					
commitment of the teachers?					
spectrum of performed procedures?					

20. How do you rate compared to before the pandemic: *
Select only one answer in a row

	Better	Comparatively	Worse
quality of conducted clinicals?			
preparation for clinicals?			
commitment to clinical work?			
time spent on preparing for clinicals?			
commitment of the teachers?			
spectrum of performed procedures?			

21. Did you feel any deficiencies in terms of diagnosing and treating patients (taking into account the current sanitary regime resulting in the limit of admitted patients)? *
Choose only one answer
Yes/No

22. If so, what kind?

Evaluation of safety during COVID-19 pandemic

23. Did you have any concerns about participating in clinical classes? *

Choose only one answer

Yes/No

24. If so, what kind?

25. On a scale from 1 to 5 (1 the lowest, 5 the highest), how would you rate the level of safety during clinical activities: *

	1	2	3	4	5
yours and the other students?					
admitted patients?					

26. In your opinion, the provided personal protective equipment is: *
Choose only one answer
Insufficient/Sufficient/Excessive

27. If insufficient or excessive, why?

28. In your opinion, should patients be tested (rapid antigen tests) before participating in clinical activities? *
Choose only one answer
Yes/No

29. In your opinion, should students and academic teachers be tested (rapid antigen tests) before participating in clinical activities? *
Choose only one answer
Yes/No

30. If so, how often?

31. In your opinion, should vaccination be mandatory for students and academic teachers before participating in clinical activities? *
Choose only one answer
Yes/No

32. Why?

Evaluation of performed blended learning in conservative dentistry with endodontics

33. On a scale from 1 to 5 (1 the lowest, 5 the highest), how would you rate the current blended-learning model? *
Choose only one answer

1	2	3	4	5

34. How would you rate the current blended-learning model compared to other dental subjects? *
Choose only one answer
Better/Comparatively/Worse

35. Briefly justify your assessment: *

36. In your opinion, is the blended-learning model beneficial and could it continue after the pandemic is over? *
Choose only one answer
Yes/No

37. Why? *

38. What do you think is the best outcome of the blended-learning model? *

39. What do you think is the worst outcome of the blended-learning model? *

Thank you for completing the survey.

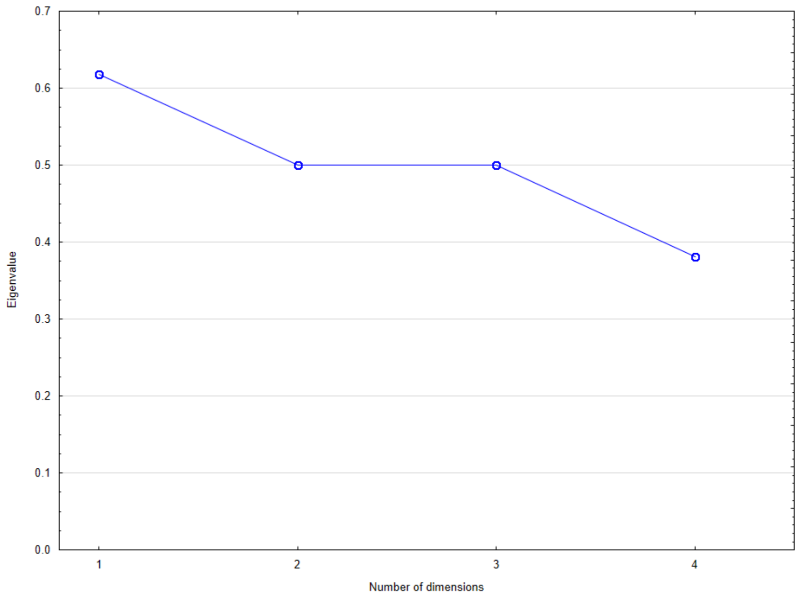

Figure S1. Scree plot for multidimensional correspondence analysis – advantages of blended learning.

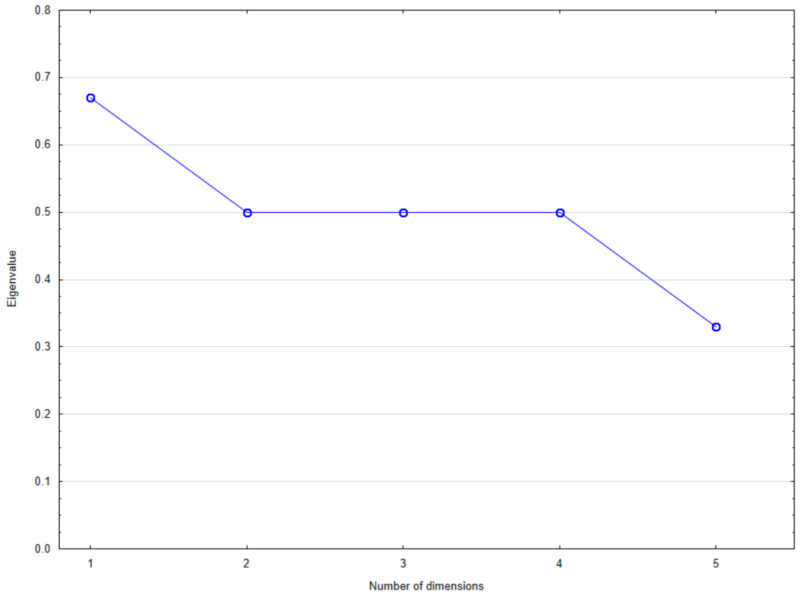

Figure S2. Scree plot for multidimensional correspondence analysis – disadvantages of blended learning.

Table S1. Parameters of determined points in multidimensional correspondence analysis – advantages of blended learning.

	x	y	Quality	Relative Inertia	x		y	
					Inertia	cos^2	Inertia	cos^2
Blended learning:pro	0.215	0.000	0.619	0.017	0.035	0.619	0.000	0.000
Blended learning:con	-2.879	$-7.30E^{-17}$	0.619	0.233	0.465	0.619	$3.70E^{-34}$	$3.98E^{-34}$
Individualised pace	0.254	-0.643	0.184	0.181	0.014	0.025	0.115	0.159
Return to recorded presentations	0.139	1.789	0.995	0.191	0.004	0.006	0.756	0.989
Time saving	-1.269	-0.382	0.585	0.188	0.325	0.536	0.036	0.049
Increased efficiency	0.906	-0.628	0.376	0.191	0.157	0.254	0.093	0.122

Table S2. Parameters of determined points in multidimensional correspondence analysis – disadvantages of blended learning.

	x	y	Quality	Relative Inertia	x		Y	
					Inertia	cos^2	Inertia	cos^2
Blended learning:pro	-0.234	0.000	0.670	0.015	0.038	0.670	0.000	0.000
Blended learning:con	2.865	$1.03E^{-17}$	0.670	0.185	0.462	0.670	$7.98E^{-36}$	$8.63E^{-36}$
virtually no drawbacks	-0.687	0.376	0.436	0.117	0.146	0.335	0.059	0.100
Reduced interpersonal contacts	0.614	0.731	0.327	0.147	0.074	0.135	0.141	0.192
Time rigour in examinations	-0.687	-2.400	0.795	0.177	0.040	0.060	0.652	0.735
Technical problems	-0.687	1.232	0.078	0.192	0.013	0.019	0.057	0.059
Decreased concentration	1.336	-0.731	0.475	0.166	0.226	0.365	0.091	0.109
